# Diverse Profiles of Ricin-Cell Interactions in the Lung Following Intranasal Exposure to Ricin 

**DOI:** 10.3390/toxins7114817

**Published:** 2015-11-17

**Authors:** Anita Sapoznikov, Reut Falach, Ohad Mazor, Ron Alcalay, Yoav Gal, Nehama Seliger, Tamar Sabo, Chanoch Kronman

**Affiliations:** Department of Biochemistry and Molecular Genetics, Israel Institute for Biological Research, Ness-Ziona 74100, Israel; E-Mails: anitas@iibr.gov.il (A.S.); reutf@iibr.gov.il (R.F.); ohadm@iibr.gov.il (O.M.); rona@iibr.gov.il (R.A.); yoavg@iibr.gov.il (Y.G.); nehamas@iibr.gov.il (N.S.); chanochk@iibr.gov.il (C.K.)

**Keywords:** ricin, lung, binding, macrophages, epithelial cells, alveolar type II cells

## Abstract

Ricin, a plant-derived exotoxin, inhibits protein synthesis by ribosomal inactivation. Due to its wide availability and ease of preparation, ricin is considered a biothreat, foremost by respiratory exposure. We examined the *in vivo* interactions between ricin and cells of the lungs in mice intranasally exposed to the toxin and revealed multi-phasic cell-type-dependent binding profiles. While macrophages (MΦs) and dendritic cells (DCs) displayed biphasic binding to ricin, monophasic binding patterns were observed for other cell types; epithelial cells displayed early binding, while B cells and endothelial cells bound toxin late after intoxication. Neutrophils, which were massively recruited to the intoxicated lung, were refractive to toxin binding. Although epithelial cells bound ricin as early as MΦs and DCs, their rates of elimination differed considerably; a reduction in epithelial cell counts occurred late after intoxication and was restricted to alveolar type II cells only. The differential binding and cell-elimination patterns observed may stem from dissimilar accessibility of the toxin to different cells in the lung and may also reflect unequal interactions of the toxin with different cell-surface receptors. The multifaceted interactions observed in this study between ricin and the various cells of the target organ should be considered in the future development of efficient post-exposure countermeasures against ricin intoxication.

## 1. Introduction

Ricin is a highly toxic ribosome-inactivating protein (RIP) derived from the seeds of the castor plant, *Ricinus communis*. Due to its high availability and to the relative ease of its production, ricin is considered as a biological threat agent [1]. Ricin is classified as a type II RIP, because it consists of an enzymatic active A-chain disulfide-linked to a B-chain. The B-chain of ricin is a galactose-specific lectin that binds to glycoproteins and glycolipids on the surface of cells [2,3]. Receptor-dependent internalization of ricin is followed by retrograde transport to the endoplasmatic reticulum, where the intra-subunit protein disulfide bond is reduced [4], and subsequently, the monomeric A-chain is released to the cytoplasm [5]. In the cytoplasm, A-chain depurinates a single adenine in the 28S rRNA. This deletion prevents the binding of elongation factor 2 to the ribosome and, therefore, is directly responsible for both the inhibition of protein translation [6] and the initiation of events leading to inflammatory responses [7,8]. Ricin toxicity is most pronounced in the case of respiratory exposures, as evidenced by the exceedingly low lethal and effective dose limits [9]. Studies in rodents and nonhuman primates have demonstrated that ricin delivered into the pulmonary system leads to acute lung injury (ALI) and symptoms resembling acute respiratory distress syndrome (ARDS) [1]. ALI is characterized by histological evidence of tissue injury, massive influx of inflammatory cells and disruption of the alveolar capillary barrier, all of which lead to lung edema and physiological dysfunction [10]. One of the key players involved in the pathogenesis and resolution of ALI/ARDS is the respiratory epithelium, which plays a role in maintaining the integrity and function of the lung. The alveolar epithelium contains two different cell types. The flat alveolar type I cells (ATI), building the structure of the alveolar wall, account for only 20% of the epithelial cells, but cover 80% of the alveolar surface area, their thin morphology allowing rapid diffusion and exchange of gases. The cuboidal alveolar type II cells (ATII), which account for 80% of the alveolar cells, secrete pulmonary surfactant, thereby lowering the surface tension and regulating fluid balance across the epithelium [11]. 

In this study, we examined the kinetics of ricin binding and internalization to lung cells *in vivo* and delineated the effects of these interactions on the cellular composition of the lung. Interactions between ricin and lung cells were examined concomitantly by both fluorescently-labeled toxin and by specific anti-ricin antibody binding. We observed differential binding patterns to various cell types *in vivo*, probably due to disparate accessibility of the different cells to the toxin and/or to the presence of different cell-type-related receptor molecules. The varied binding performances of the different cell types comprising the mouse lung were accompanied by differential alterations in the hematopoietic and parenchymal cell populations. Taken together, our data suggest that differential ricin binding and internalization determine the impairment of tissue integrity and function. 

## 2. Results

### 2.1. Differential Binding of Ricin to the Cells of the Mouse Lung 

**Figure 1:**
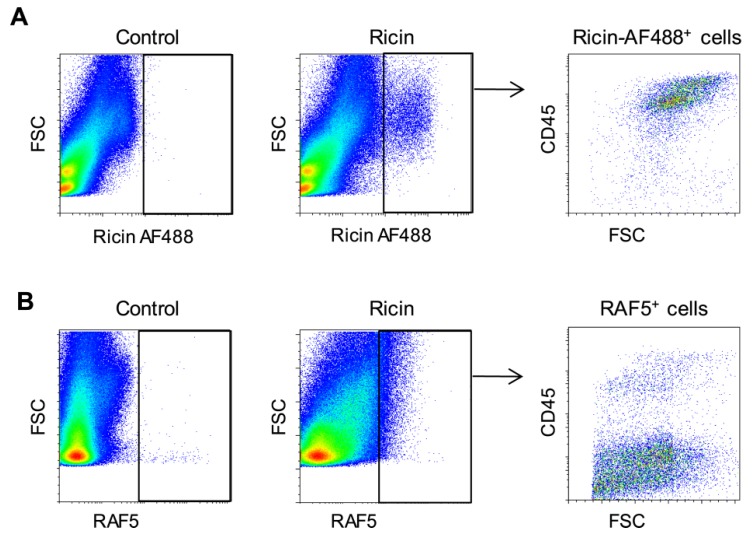
*In vivo* binding of ricin to cells of the mouse lung. Lung cells isolated 3 h after intranasal exposure to ricin-AF488 were analyzed by fluorescence activated cell sorting (FACS) for bound toxin direct toxin-fluorescence (**A**) or by staining of the cells with anti-ricin antibody, RAF5 (**B**).

To characterize the interactions between ricin and cells of the lung, mice were intranasally exposed to fluorescently-labeled ricin at a lethal dose of 2 LD_50_. We monitored toxin-associated cells by examining the forward scatter (FSC) profile of pulmonary cells isolated 3 h after intoxication. Intoxication led to the labeling of a defined population of cells, mainly of hematopoietic (CD45^+^) origin (Figure 1A). Since fluorescent toxin identifies both internal and external cell-associated toxin, ricin binding was monitored by an additional technique, utilizing purified anti-ricin antibody, RAF5, for tagging the toxin. It should be noted that when this technique is employed, the detection of cell-associated ricin reflects a primary interaction between the antibody and the toxin, which occurred at the cell exterior. Indeed, monitoring toxin-associated cells by this technique revealed a distinctly different pattern, attesting to the binding of the toxin mainly to parenchymal (CD45^−^) cells (Figure 1B). Identification of ricin binding to CD45^+^ cells mostly by toxin fluorescence and not by antibodies indicates that in these cells, the bulk of cell-associated toxin has already been internalized. In contrast, ricin-bound CD45^−^ cells were mainly detectable by antibodies, indicating that in these cells, ricin was associated with the exterior surface of the cells. Toxin/cell interactions in CD45^−^ cells could not be observed by the direct fluorescent-toxin monitoring technique, suggesting that this technique is less sensitive than the antibody-tagging technique. 

**Figure 2:**
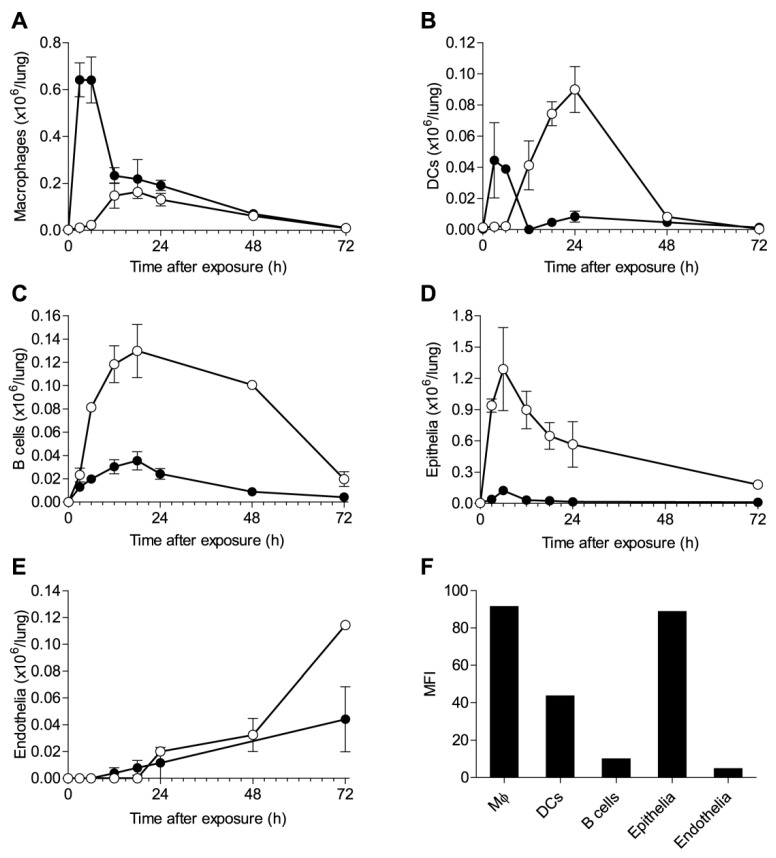
Kinetics of ricin binding to individual cell populations in the lungs. Mice were intranasally exposed to ricin-AF488 and lung cells isolated at 3, 6, 12, 18, 24, 48 and 72 h were analyzed by FACS for ricin binding, by detection of AF488^+^ cells (filled circles) or by staining with anti-ricin RAF5 (open circles) (six mice per group). Quantification by AF488 and RAF5 of ricin-bound MΦs (**A**), DCs (**B**), B cells (**C**), epithelial cells (**D**) and endothelial cells (**E**). (**F**) Mannose receptor expression in various cells as measured by FACS. Values are displayed as mean fluorescence intensity (MFI).

These findings prompted us to determine the kinetics of ricin binding to individual cell populations of the lung. Mice were intoxicated with fluorescently-labeled ricin, and lung cells isolated at different time points were then stained for anti-ricin antibodies and analyzed for ricin binding. In MΦs and DCs, as gated in Figure S1A, two temporally-distinct peaks were detected by the two techniques (Figure 2A,B). Whilst peak binding at 3–6 h after intoxication was detected by fluorescent toxin, labeling with antibodies identified a later peak, at 18 h after intoxication. The failure of the sensitive antibody-based technique to detect toxin-associated cells at the earlier time point indicated that the peak binding at this time point relates to cells that have already internalized the toxin. However, the ratio between the early and late peaks of toxin association was different in the two cell types. In the case of MΦs (Figure 2A), the greater amount of toxin was internalized at the early time point, while in the case of DCs (Figure 2B), most of the association of toxin with the cells occurred at the late time point, remaining bound to the cell exterior. Early binding of ricin by MΦs preceded that of DCs, while significant binding to MΦs was detected already 1 h after intoxication; binding to DCs was observed at 3 h after exposure (Figure S2A–C). The determination of the binding profile to B cells (Figure S1B) identified a single late peak at 18 h after intoxication by both techniques (Figure 2C), while toxin binding to neutrophils (Figure S2A) could not be detected at all (Figure S2D). Analysis of toxin interactions with parenchymal cells demonstrated that binding to epithelial cells (Figure S1C) is characterized by a single peak detected by anti-ricin antibodies at an early time point of 6 h (Figure 2D). In contrast, ricin binding to endothelial cells (Figure S1C) was discernable only at 24 h after intoxication and did not reach peak levels within the time frame of these experiments (Figure 2E). The spatial localization of the different cells types within the lungs could play a role in the bi-phasic shaping of the ricin binding profile. Following pulmonary intoxication, ricin first encounters the MΦs located in the alveolar lumen and the DCs protruding through the epithelial network, and only then, the toxin has access to the interstitium. However, the fact that a single cell type may display both early and late binding peaks, as in the case of MΦs and DCs, suggests that the composite binding patterns are also an outcome of different mechanisms of binding. The B-chain of ricin is a galactose-specific lectin that binds to glycoproteins or glycolipids at the cell surface [2,3]; yet, in addition to this canonical route, ricin can enter cells through a secondary route, via the mannose receptor that is present on MΦs [12]. This receptor binds to high mannose oligosaccharide chains present on both A and B subunits of the toxin and is more efficient in delivering ricin to the cytoplasm [13]. To evaluate the possible role of this alternative binding pathway, mannose receptor density was measured for different cell types of the lung (Figure 2F). In both MΦs and DCs, the presence of this highly efficient receptor could explain the early peak of ricin binding and the measured difference in receptor density between the two cell types, correlating well with the relative amounts of toxin that were associated with these cells (Figure 2A,B). Epithelial cells were found to express the mannose receptor at levels that did not fall from those of MΦs (Figure 2F) and, indeed, displayed a high early binding peak (Figure 2D), while B cells and endothelial cells, both of which were devoid of the mannose receptor (Figure 2F), did not display an early peak of binding (Figure 2C,E). Toxin association via galactose, which is less efficient than via the mannose receptor, could be responsible for the later peaks of binding observed in the different cell types. 

### 2.2. Alterations in Lung Cell Populations Following Ricin Intoxication

**Figure 3:**
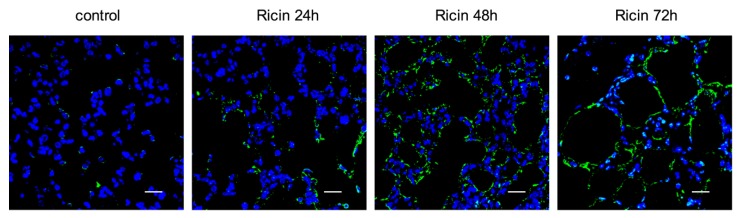
Measurement of apoptotic changes in the lung after ricin intoxication. Apoptotic staining (TUNEL, green; DAPI, blue) in naive (left) and non-labeled-ricin-intoxicated mice (six mice per group). Scale bars indicate 20 µm.

Our findings show that ricin binds to cells of the lung in a differential manner, perhaps through different receptors. Upon entry to the cytosol, ricin inhibits protein synthesis by the irreversible inactivation of ribosomes, which in turn leads to cell death. Indeed, massive apoptotic changes were readily detected in the lung tissue at late time points after intoxication (24–72 h; Figure 3). In contrast, apoptosis could not be detected at early time points, suggesting that cell elimination early after intoxication, such as that displayed for MΦs and DCs, stems from necrotic death or pyroptosis. 

**Figure 4:**
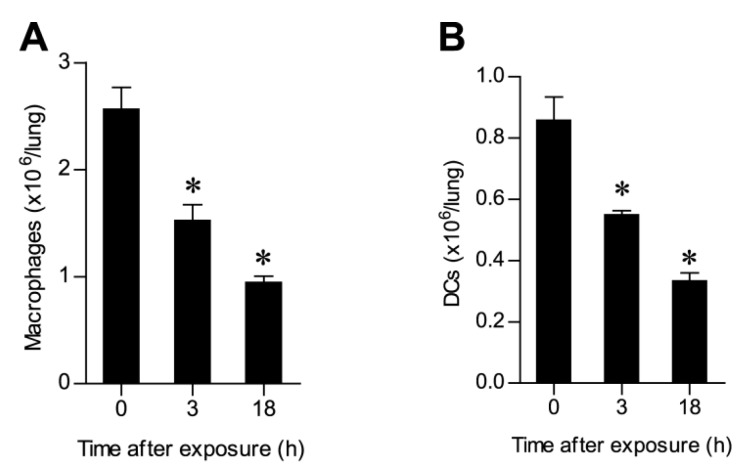

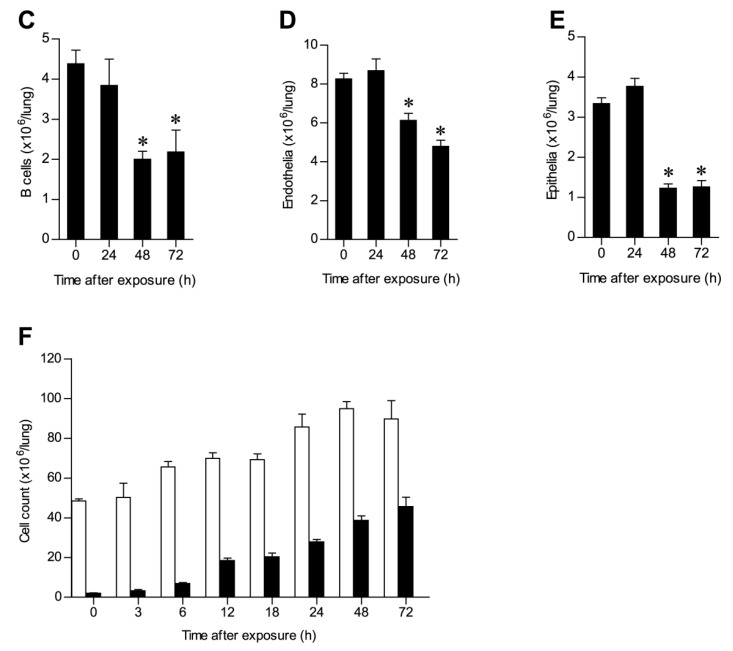
Cell counts at different time points following ricin intoxication. Lung cells isolated from mice at different time points after intranasal exposure to ricin-AF488 and then stained for different cell surface markers were analyzed by FACS (five to ten mice per group). (**A**) MΦs, (**B**) DCs, (**C**) B cells, (**D**) endothelial cells, (**E**) epithelial cells and (**F**) total lung cells (open bars) and neutrophils (filled bars); * *p* < 0.05.

To appreciate the correlation between binding kinetics and alterations in the lung cell populations, we performed a quantitative analysis of pulmonary cells following ricin intoxication. Evaluation of the alterations in MΦs allowed us to determine that a two-fold reduction occurred as early as 18 h after intoxication (Figure 4A) in line with our finding that ricin binding to this cell type occurred early (Figure 2A). Likewise, a two-fold reduction in the DCs population could be discerned already at 18 h after intoxication (Figure 4B). Profiling of the B cell and endothelial cell populations, both of which were found to bind toxin at late time points (Figure 2C,E), demonstrated that a two-fold reduction in these cells was reached at 48 h and 72 h after intoxication, respectively (Figure 4C,D). In the case of epithelial cells, although binding of toxin occurred early after intoxication (Figure 2D), a significant decrease in their number was evidenced only later: epithelial cell counts dropped from (3.3 ± 0.8) × 10^6^ down to (1.2 ± 0.5) × 10^6^ at 48 h after intoxication (Figure 4E). In spite of the cytotoxic effect of ricin and the marked elimination observed for various cell types, the overall number of cells comprising the lungs increased over time. Thus, at 6 h after intoxication, the number of lung cells (48.5 ± 6) × 10^6^ increased to (65.6 ± 6) × 10^6^, while 48 h after intoxication, numbers reached (95 ± 13) × 10^6^ cells (Figure 4F). This striking rise in cell number correlated with the concomitant recruitment of neutrophils to the lungs. Neutrophil counts (~2 × 10^6^ cells) were tripled 6 h after exposure to ricin, and at 48–72 h after intoxication, the size of this cell population was nearly equal to the number of cells comprising the entire lungs in naive mice (Figure 4F). 

### 2.3. Ricin Intoxication Results in Specific Loss of Alveolar Type II Epithelia

**Figure 5:**
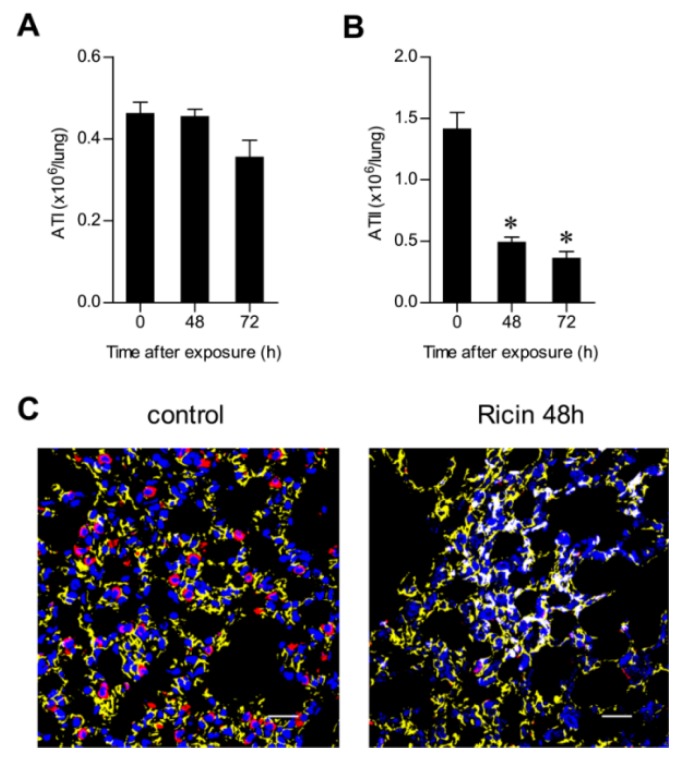
Effect of exposure to ricin on alveolar type I (ATI) and ATII cells. Lungs were harvested at different time points after intranasal exposure to unlabeled ricin. (**A**) ATI and (**B**) ATII cells were analyzed by FACS 48 and 72 h after intoxication (five mice per group). * *p* < 0.05. (**C**) Immunofluorescence analysis of pro-surfactant C (pro-SPC) (red), T1α (yellow) and DAPI (blue) staining of lung tissue from naive (left) and 48 h post-intoxication (right) mice. Scale bars indicate 20 µm.

**Figure 6:**
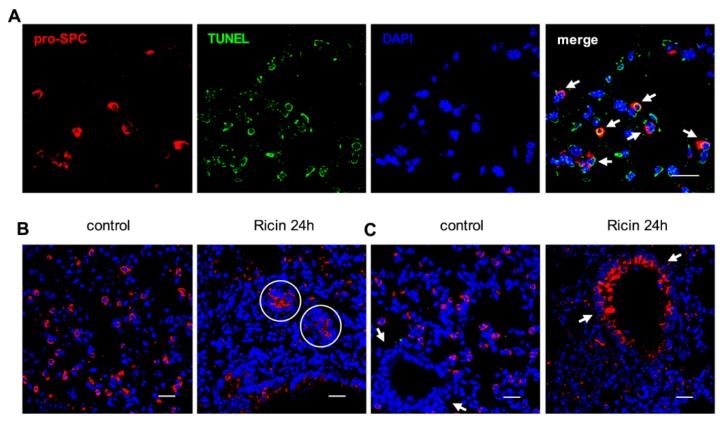
Immunofluorescence analysis of ATII cells after ricin exposure. (**A**) Co-immunofluorescence for pro-SPC (red), TUNEL (green) and DAPI (blue) from lung tissue 24 h after intoxication. Arrows point to TUNEL-positive, apoptotic ATII cells; (**B**) Immunofluorescence for pro-SPC (red) and DAPI (blue) staining of lung tissue from naive (left) and 24 h post-intoxicated (right) mice. Circles indicate pro-SPC^+^ ring structures; (**C**) Visualization of bronchiole structures (arrows) stained for pro-SPC (red) and DAPI (blue) in lung tissue from naive (left) and 24 h post-intoxicated (right) mice. Scale bars indicate 20 µm.

Lung alveolar epithelial cells are of two types, respiratory alveolar type I (ATI) and surfactant-secreting alveolar type II (ATII) cells [14], the latter being dispersed between the ATI cells. ATI and ATII cells can be differentiated by specific labeling of podoplanin (T1α) [15] and pro-surfactant C (pro-SPC) [16], respectively. FACS analysis demonstrated that ATII cells (CD45^−^, CD31^−^, pro-SPC^+^), but not ATI cells (CD45^−^, CD31^−^, T1α^+^) (Figure S3), were depleted from the intoxicated lung at 48 h after exposure (Figure 5A,B). This unexpected finding, that epithelial elimination in the intoxicated lung was restricted to ATII cells, was also manifested by immunohistochemical analysis (Figure 5C). TUNEL staining for apoptosis was prevalent in pro-SPC labeled cells, suggesting that apoptosis of ATII cells precedes their elimination (Figure 6A). It should be mentioned that at 24 h after ricin intoxication, but not at later time points, pro-SPC^+^ ring structures were detected in the damaged parenchyma (Figure 6B). Moreover, pro-SPC expression, undetected in the bronchial epithelia of naive lungs, was readily visualized in the bronchial epithelia of intoxicated lungs (Figure 6C). This incongruous *de novo* expression of the surfactant precursor presumably reflects an attempt to restore pulmonary functionality by means of synthesizing surfactant.

## 3. Discussion

In the present study, we monitored the interactions between ricin and the cells of the lung and delineated the alterations occurring to different pulmonary cell populations following *in vivo* exposure to the toxin. Over the years, various studies examined the *in vitro* binding of ricin to isolated human or murine cell cultures [17,18]. Such studies cannot faithfully reflect processes occurring after *in vivo* intoxications, since they do not take into account pertinent factors, such as the anatomical structure of the target organ, the actual location of different cells within the organ and differences in cell accessibility to the toxin. 

Here, we show that following intranasal exposure of mice to ricin, the toxin binds mainly and in a rapid manner to mononuclear myeloid cells, *i.e.*, MΦs and DCs. Our data support the concept that alveolar MΦs and DCs serve as early target cells and, thus, may play a critical role in ricin-induced ALI and inflammation. Indeed, it has been shown by others that *in vivo* depletion of MΦs prior to ricin delivery to the pulmonary system of mice resulted in diminished inflammatory signs, including reduced neutrophilia and pulmonary edema [19]. Moreover, alveolar MΦs were shown to be attached to the alveolar epithelium and to conduct immunosuppressive intercommunications with epithelial cells by Ca^2+^ waves. During inflammation, necrosis of MΦs promotes neutrophil recruitment and secretion of pro-inflammatory cytokines [20]. 

Comprehensive analysis of ricin binding and cytotoxic kinetics allowed us to observe important phenomena regarding the mode of action of ricin. One key observation is the multiphasic nature of ricin binding: some cells bind the toxin at an early time point, others significantly later, while a third group of cells displays binary binding to the toxin, both at the early and late stages. An additional observation of importance is that different cell types, which bind the toxin at the same time, are eliminated from the lungs at different rates. This complex pattern of cell/toxin interactions can be explained by the differential accessibility of various types of cells to the toxin, as well as by the presence of different toxin receptors at the surface of the cells. Indeed, MΦ, dendritic and epithelial cells, which were found to express the mannose receptor, all bound the toxin early after intoxication. Studies carried out by others implicated the importance of ricin uptake by mannose receptor-expressing MΦs, in scavenging and degradation of the toxin [21]. In the present study, we demonstrate that other cell types, such as the alveolar epithelial cells, also express the mannose receptor and bind the toxin early after intoxication and, therefore, may also play a role in controlling intoxication severity. Although myeloid and epithelial cells bind toxin early after intoxication, these cells differed radically in their rate of elimination. Mononuclear myeloid cells, which rapidly internalized ricin at high quantities, as attested by their ready detection by fluorescent-labeled toxin, but not by antibody, were eliminated early after intoxication. In the case of DCs, following early rapid internalization of ricin, a late peak of toxin association with the outer surface of the DCs was observed. It may well be that a subset of DCs that expresses the mannose receptor internalizes the toxin rapidly, precipitating their elimination from the lungs. The toxin then binds to another subset of DCs that is devoid of the mannose receptor, whereby internalization is relatively slow, leading to the detection of the bound toxin at the cell exterior. 

Elimination of epithelial cells was observed late after intoxication, even though these cells bound the toxin as early as the myeloid cells. This may be due to different rates of internalization or intracellular trafficking of ricin to the cell cytosol. In addition, it may well be that the two cell types differ in the extent of retro-endocytosis of the toxin. Retro-endocytosis is a well-documented process, by which ricin is actively shuttled out of the cell, before it has reached the cytosol, where it could inactivate ribosomes [22]. Indeed, it has been demonstrated that the extent of retro-endocytosis, and not the rate of toxin uptake, serves in some instances as the decisive factor in determining the comparative toxicity of different type II RIP toxins [23]. Whether a single toxin, *i.e.*, ricin, is expelled at different rates by different cell types, has, to the best of our knowledge, not yet been addressed. 

Resolving the alveolar epithelia to two functional subtypes allowed us to determine that ricin specifically and significantly damaged ATII, and not ATI, cells. This preferential injury is opposed to the accepted notion that ATI cells are more susceptible to injury and death in various scenarios of lung insult [11]. It is unlikely that the reduction in ATII cells observed stems from their differentiation to ATI cells, since the transition from ATII to ATI cells occurs in reaction to a reduction of the ATI cell population. Such a reduction was not observed in this study. Preferential injury of ATII cells has been also observed in the case of influenza virus infection and bleomycin treatment in mice models. Following ATII cell eradication in response to these agents, bronchiolar epithelial cells differentiate to replenish the ATII cell population [24]. Interestingly, we observed that prior to ATII cell loss in ricin-intoxicated lungs, bronchiolar epithelial cells transiently express the ATII-specific pro-SPC marker. It is tempting to surmise that this phenomenon relates to an attempt to rehabilitate ATII function. However, this attempt turned out to be abortive, assumingly due to the damage of these cells later on by the toxin. 

The interaction of ricin with the pulmonary endothelial cells resulted in the elimination of approximately one third of the cells by 72 h after intoxication. Endothelial cell damage is known to promote increased permeability and movement of extravascular fluid to the lungs and is a hallmark of ARDS [25]. One may assume that this insult intensifies the severe lung edema and alveolar flooding caused by the damage to epithelial cells.

Endothelial and epithelial injury was accompanied by an influx of neutrophils. Overzealous activation of neutrophils can lead to tissue damage and prolonged inflammation by the release of cytotoxic and immune cell-activating agents, such as proteinases, cationic polypeptides, cytokines and reactive oxygen species [26]. Indeed, neutrophils are considered to play a key role in the progression of ALI and ARDS [27]. In a mouse model of ARDS, the number of neutrophils in the bronchoalveolar lavage fluid correlates with the severity of the lung injury and disease outcome, whereas neutrophil depletion assuages lung impairment [28]. Here, we show that neutrophils, which were massively recruited to the lungs following exposure to ricin, failed to bind the toxin. Although it has been shown by others that human polymorphonuclear cells bind ricin in cell culture [17], *in vitro* toxin-cell interactions do not necessarily reflect, in a faithful manner, the processes occurring in the more discriminative *in vivo* setup. The refractivity of neutrophils, which are present at exorbitant numbers in the intoxicated lung, towards ricin may enhance lung deterioration due to the nefarious activities of these cells.

Previous studies carried out at our laboratory [29,30] have probed the interrelationship between ricin-induced protein synthesis arrest and the clinical manifestation of ricin intoxication, namely the onset of a severe edematous inflammation accompanied by massive recruitment of neutrophils and cytokine storming. The present study underlines the intricate nature of the varied interactions of ricin with the cells of the lung. We show that the toxin interacts rapidly with cell types normally involved in innate immune responses and plays key roles in the onset and maintenance of inflammation, as well as with parenchymal cells responsible for the primary function of the lungs. The development of an efficient therapeutic countermeasure against ricin exposure should take into consideration the multifaceted interactions between this noxious toxin and the various cells populating the target organ. 

## 4. Experimental Section

### 4.1. Animal Studies

Animal experiments were performed in accordance with the Israeli law and approved by the Ethics Committee for animal experiments at Israel Institute for Biological Research. Treatment of animals was in accordance with regulations outlined in the USDA Animal Welfare Act and the conditions specified in the Guide for Care and Use of Laboratory Animals (National Institute of Health, 1996). Mice were female CD-1 (Charles River Laboratories Ltd., Margate, UK) weighing 27–32 g. 

### 4.2. Fluorescent Ricin Labeling and Intoxication

Ricin was purified as described [29] and conjugated (1 mg) with the Alexa Fluor^®^ 488 protein labeling kit (Molecular Probes, Thermo Fisher Scientific, Waltham, MA, USA) according to the manufacturer’s instructions. The cytotoxicity of labelled toxin (~5 dye/protein (mol/mol)) was determined in a cell-based assay developed in the past [31]. Briefly, labeled ricin was added to HEK-293 cell cultures, which secrete the enzyme acetylcholinesterase (AChE) in a constitutive manner. Secreted AChE was measured in the cell growth medium at 18 h post-exposure, and activity levels were compared to those measured for non-labelled ricin. This allowed us to determine a ~2-fold reduction in toxicity of labeled ricin. Intranasal intoxication with ricin at a dose of 2LD_50_ (unlabeled and labeled ricin, 7 and 14 µg/kg, respectively, diluted in PBS) was performed after anesthetizing mice i.p. with ketamine (1.9 mg/mouse) and xylazine (0.19 mg/mouse). The total volume of toxin, 50 µL, was slowly applied (25 µL/nostril) using a gel-loading tip (×0.6 mm, USA Scientific, Orlando, FL, USA).

### 4.3. Purification of Anti-Ricin Specific Antibody RAF5

Ricin was coupled to an affinity column (AminoLink^®^ kit, Pierce Biotechnology, Waltham, MA, USA) according to the manufacturer’s instructions. Rabbit anti-ricin hyperimmune sera were loaded on to the column. Following incubation (1 h), the column was washed (PBS), and the specific antibody was eluted (50 mM glycine-HCl, pH 2.7, 10% ethylene glycol). The antibody concentration was 1.3 mg/mL, and K_D_ = 1 nM (Octet^red^). For RAF5 staining, cells were incubated on ice for 15 min with the purified RAF5 antibody, then washed and stained for an additional 15 min with secondary donkey anti-rabbit allophycocyanin (APC)-labeled antibody (Jackson ImmunoResearch, West Grove, PA, USA). To avoid internalization of the stained ricin with RAF5 into the cells, during sample processing and staining, NaN_3_ was added to the buffers (final concentration = 0.05%), and samples were kept on ice during the whole experiment. 

### 4.4. FACS Analysis 

Lungs harvested and cut into small pieces were digested (2 h, 37 °C) with 4 mg/mL collagenase D (Roche, Mannheim, Germany). Tissue was meshed through a 40-µm cell strainer, and RBCs were lysed. Cells were stained using antibodies coupled to: phycoerythrin (PE) (anti-Gr-1 (RB6-8C5), I-A/I-E (M5/114.15.2), CD19 (1D3), CD31 (PECAM-1, 390)); allophycocyanin (APC) (anti-CD11c (N418), CD326 (EpCAM, G8.8)); peridinin chlorophyll protein-Cy5.5 (PerCP-Cy5.5) (F4/80 (BM8)); biotinylated (anti-CD45 (30-F11)); Alexa Fluor 488 (anti-podoplanin (T1alpha, 8.1.1)) and purified polyclonal anti-pro-SPC (Millipore, Temecula, CA, USA) followed by donkey anti-rabbit IgG (H+L) coupled to APC (Jackson ImmunoResearch, West Grove, PA, USA). Biotinylated antibodies were followed by staining with PerCP-Cy5.5-labeled streptavidin. Cells were defined as: neutrophils (CD11c^−^, Gr-1^high^); alveolar MΦs (autofluorescent, CD11c^high^, Gr-1^int^, F4/80^+^, MHCII^int^); DCs (CD11c^int^, Gr-1^−^, F4/80^−^, MHCII^high^); B cells (CD19^+^); endothelial cells (CD45^−^, CD31^+^); and epithelial cells (CD45^−^, CD326^+^). For mannose receptor staining, cells were stained for biotinylated-anti-CD206 (C068c2). Unless otherwise indicated, the reagents were obtained from BioLegend (San Diego, CA, USA) and eBioscience (San Diego, CA, USA). Cells were analyzed on FACSCalibur (BD Biosciences, San Jose, CA, USA) using FlowJo software (version 7.1.2, Tree Star, Ashland, OR, USA). The number of each cell type was calculated by multiplying the percentage of a specific cell population (as gated and determined by FACS) by the total lung cell count.

### 4.5. Immunohistochemistry

Lungs perfused with PBS were harvested and immersed in neutral buffered formalin (4%, 2 weeks, room temperature) prior to embedding. Sections (5 µM) were mounted on glass slides and deparaffinized. Antigen retrieval was performed by incubation in Target Retrieval Solution (DAKO, 30 min, 95 °C). After blocking in 5% BSA in PBS, slides were incubated (overnight, 4 °C) with purified polyclonal anti-pro-SPC (Millipore, Temecula, CA, USA) and anti-podoplanin (T1alpha, 8.1.1, eBioscience, San Diego, CA, USA). Staining with primary antibodies was followed by Alexa Fluor 594-coupled donkey anti-rabbit and FITC-coupled goat anti-Armenian hamster antibodies (Molecular probes, Eugene, OR, USA), respectively. For nuclear staining, slides were mounted with Prolong^®^ Gold antifade reagent containing DAPI (Molecular probes, Eugene, OR, USA). For apoptotic staining [32], slides were incubated with 20 µg/mL proteinase K diluted in TE buffer (30 min, 37 °C) and then stained for TUNEL (Serotec, Oxford, UK) according to the manufacturer’s instructions. Analysis was performed using an LSM 710 confocal microscope (Carl Zeiss, Oberkochen, Germany) equipped with the following lasers: argon multiline (458/488/514 nm), diode 405 nm, DPSS 561 nm and helium-neon 633 nm.

### 4.6. Statistical Analysis

Two-tailed *t*-tests were used to compare groups. Significance was set at *p* < 0.05. Analysis was performed using GraphPad Prism. Data are represented as the means ± SEM.

## Supplementary Materials

Supplementary materials can be accessed at: http://www.mdpi.com/2072-6651/7/11/4817/s1.
