# Supplementary material for: Diverse Profiles of Ricin-Cell Interactions in the Lung Following Intranasal Exposure to Ricin
Source: Toxins (Basel). 2015 Nov 17;7(11):4817–31. doi: 10.3390/toxins7114817 (PMC4663535; doi:10.3390/toxins7114817)
Supplement: Supplementary file 1 [file toxins-07-04817-s001.pdf]

Supplementary Information

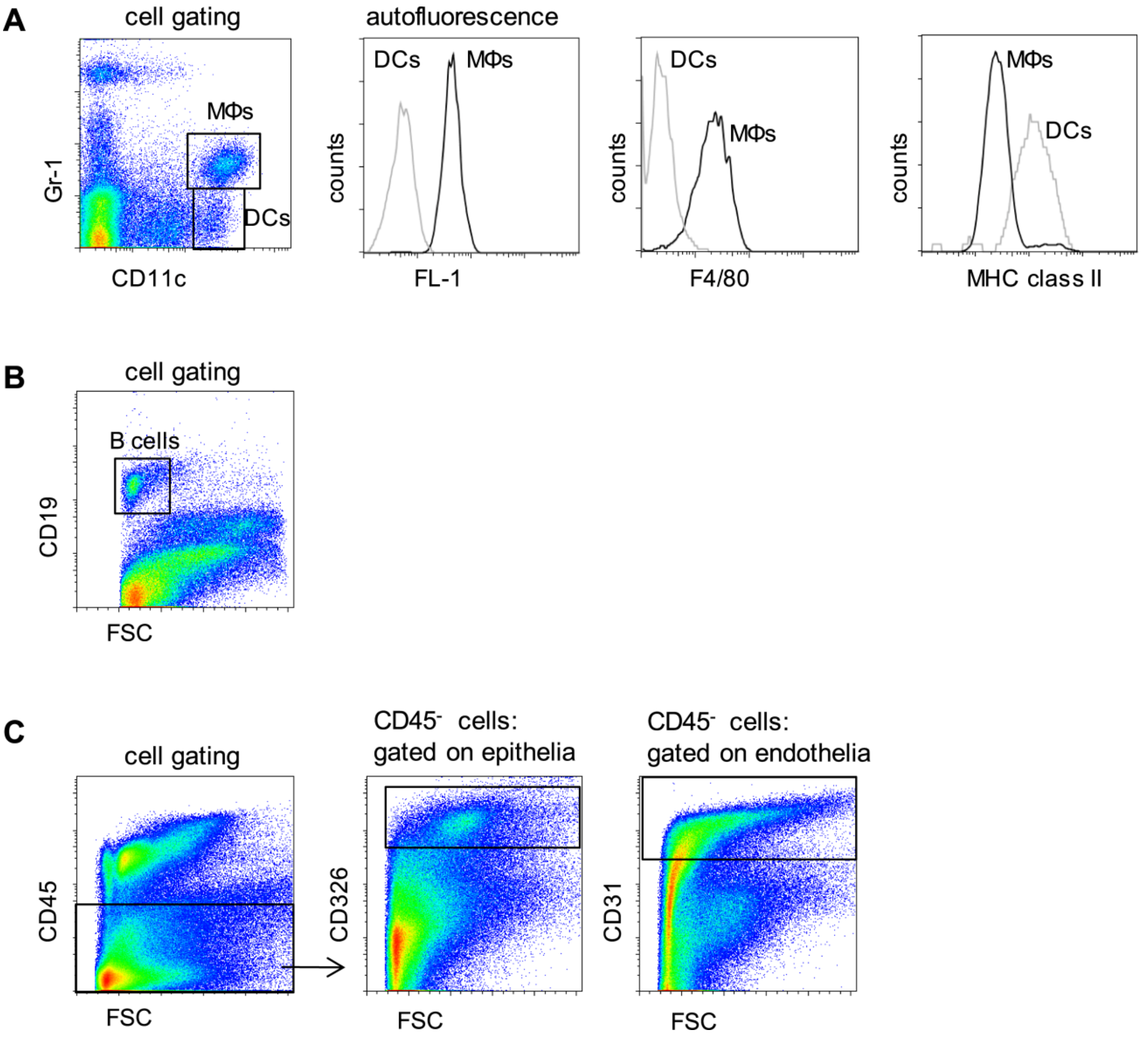

Figure S1. FACS analysis of cell populations in the lung. (**A**) Representative dot plot for gating alveolar MΦs (CD11c^{high}, Gr-1^{int}) and DCs (CD11c^{int}, Gr-1$^{−}$). Alveolar MΦs were autofluorescent as shown by FL-1 and the expressed F4/80 marker. DCs expressed higher levels of major histocompatibility complex (MHC) class II than MΦs; (**B**) Representative dot plot for gating B cells (CD19^{+}); (**C**) Representative dot plots for gating epithelia (CD45$^{−}$, CD326^{+}) and endothelia (CD45$^{−}$, CD31^{+}).

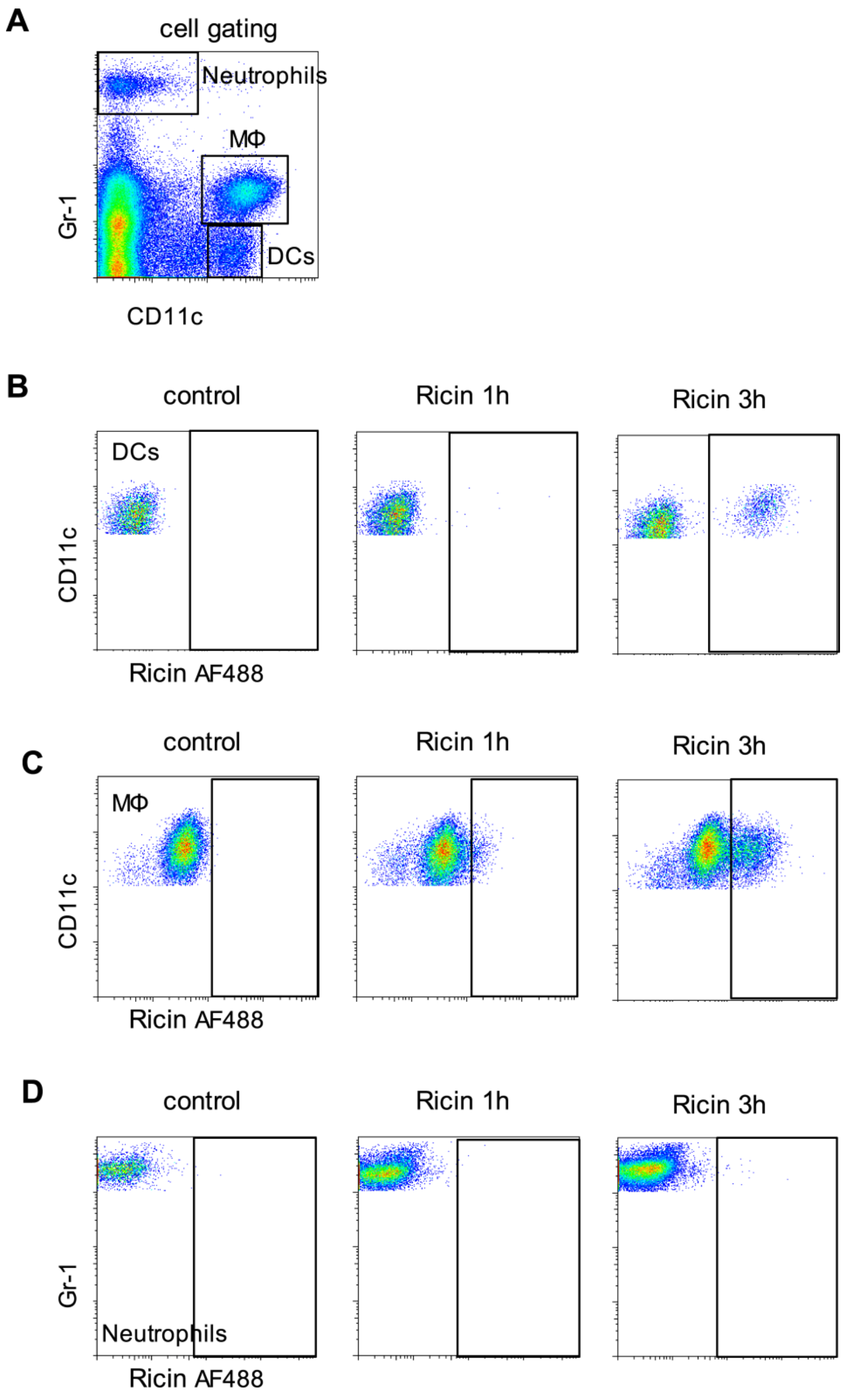

Figure S2. FACS analysis of early fluorescent ricin binding in cell populations in the lung. (**A**) Representative dot plot for gating neutrophils (CD11c^{-}, Gr-1^{high}), alveolar autofluorescent MΦs (CD11c^{high}, Gr-1^{int}) and DCs (CD11c^{int}, Gr-1^{-}). Lung cells isolated after 1 or 3 h from mice intranasally-exposed to ricin-AF488 were analyzed for ricin-bound DCs (**B**), MΦs (**C**) and neutrophils (**D**).

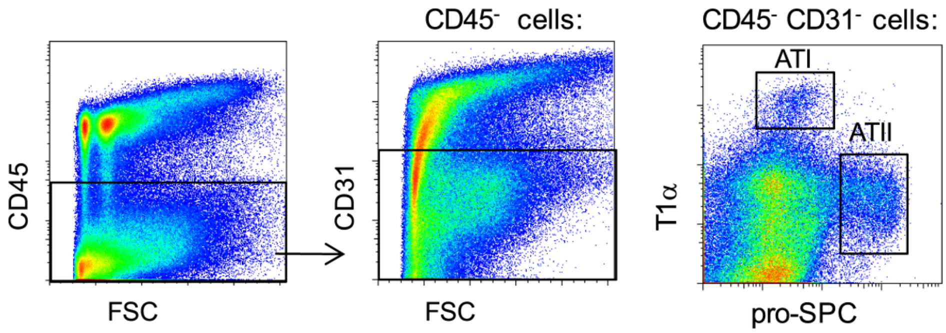

Figure S3. FACS analysis of lung epithelial subpopulations. ATI cells were gated as $CD45^{-}$, $CD31^{-}$, $T1^{+}$ and pro-SPC^{-}, and ATII cells were gated as $CD45^{-}$, $CD31^{-}$, $T1^{-}$ and pro-SPC^{+}.
